# Biodegradable nanoparticles for the treatment of epilepsy: From current advances to future challenges

**DOI:** 10.1002/epi4.12567

**Published:** 2021-12-13

**Authors:** Lorena Bonilla, Gerard Esteruelas, Miren Ettcheto, Marta Espina, María Luisa García, Antoni Camins, Eliana B. Souto, Amanda Cano, Elena Sánchez‐López

**Affiliations:** ^1^ Department of Pharmacy Pharmaceutical Technology and Physical Chemistry Faculty of Pharmacy and Food Sciences University of Barcelona Barcelona Spain; ^2^ Institute of Nanoscience and Nanotechnology (IN2UB) University of Barcelona Barcelona Spain; ^3^ Centre for Biomedical Research in Neurodegenerative Diseases Network (CIBERNED) Carlos III Health Institute Madrid Spain; ^4^ Department of Pharmacology, Toxicology and Therapeutic Chemistry Faculty of Pharmacy and Food Sciences University of Barcelona Barcelona Spain; ^5^ Institute of Neurosciences University of Barcelona Barcelona Spain; ^6^ Department of Pharmaceutical Technology Faculty of Pharmacy, University of Coimbra Coimbra Portugal; ^7^ CEB – Centre of Biological Engineering University of Minho Braga Portugal; ^8^ Ace Alzheimer Center Barcelona – Universitat Internacional de Catalunya Barcelona Spain

**Keywords:** epilepsy, lipid nanoparticles, nanomedicine, nanotechnology, neurodegenerative diseases, polymeric nanoparticles

## Abstract

Epilepsy is the second most prevalent neurological disease worldwide. It is mainly characterized by an electrical abnormal activity in different brain regions. The massive entrance of Ca^2+^ into neurons is the main neurotoxic process that lead to cell death and finally to neurodegeneration. Although there are a huge number of antiseizure medications, there are many patients who do not respond to the treatments and present refractory epilepsy. In this context, nanomedicine constitutes a promising alternative to enhance the central nervous system bioavailability of antiseizure medications. The encapsulation of different chemical compounds at once in a variety of controlled drug delivery systems gives rise to an enhanced drug effectiveness mainly due to their targeting and penetration into the deepest brain region and the protection of the drug chemical structure. Thus, in this review we will explore the recent advances in the development of drugs associated with polymeric and lipid‐based nanocarriers as novel tools for the management of epilepsy disorders.

**Key Points**

- Nanomedicine is a promising tool for central administration of drugs.
- Biodegradable nanoparticles can enhance therapeutic efficacy of antiseizure medications and offer a prolonged drug release.
- Surface functionalization of biodegradable nanoparticles could contribute to enhance their permeability trough the BBB.
- Polymeric and lipid nanoparticles constitute promising strategies in order to improve pharmacokinetics of antiseizure medications.

## 1 INTRODUCTION

Epilepsy is a chronic disease of the central nervous system (CNS) mainly characterized by an abnormal electrical activity of the neurons of hippocampus and cortex regions. According to current data from the World Health Organization (WHO), epilepsy affects more than 50 million people worldwide, and each year approximately 5 million new cases are diagnosed.^1^, ^2^ The incidence of epilepsy is higher in low‐income countries, being 139 cases per 100 000, while in developed countries it is approximately 50 cases per 100 000 inhabitants.^3^

Among the high prevalent and disabling neurological diseases, epilepsy is considered one of the most serious disorders nowadays.^4^ In this way, epilepsy is defined as a chronic alteration of the CNS characterized by an imbalance in neuronal electrical activity that gives rise to different recurrent and unpredictable seizure events that, depending on the severity, can lead to neuronal death of certain brain areas.^5^ Seizures are the result of the electric shocks produced during the outbreak and can involve either part of the body (partial) or the whole body (generalized) and be accompanied by loss of consciousness and control of bowel function. They can vary in their frequency and intensity and in turn are conditioned by the brain area where they originate, as well as the natural variability between individuals. A large amount of death causes related to epilepsy are due to falls, drowning, burns, and prolonged seizures that are not properly addressed.^6^

Importantly, seizures and epilepsy are two different terms. In 2002, the International League Against Epilepsy (ILAE) wrote a glossary of descriptive terminology, where it was defined: (i) “Epileptic seizure,” a transitory occurrence of signs and/or symptoms due to excessive neuronal activity or an abnormal synchronicity in the brain; (ii) “Epilepsy,” a brain disorder characterized by a long‐lasting predisposition to generate epileptic seizures and by the neurobiological, cognitive, psychological, and social consequences of this condition. The definition of epilepsy requires the occurrence of at least one epileptic seizure, and finally (iii) “Epileptic syndrome,” a complex of signs and symptoms that define a single epilepsy condition. This must involve more than one type of seizure; therefore, frontal lobe seizures per se, for example, do not constitute an epileptic syndrome.^7^

Its classification is very complex, since there are different considerations depending on the seizure focus, age of onset or concomitant diseases, among others. In general, WHO classifies epilepsy as idiopathic or secondary epilepsies.^2^ Idiopathic epilepsies are the epileptic syndromes whose cause is not identifiable. Conversely, secondary or symptomatic epilepsies are characterized by being a consequence derived from another disorder, such a brain tumor, brain infections, or cranial traumatisms. Recently, the ILAE more broadly classified epilepsies according to their etiology: (i) structural origin, which refers to visible abnormalities, identifiable by neuroimaging techniques, in brain structures, and which in turn differentiates between acquired and genetic; (ii) genetic origin, due to a known or unknown genetic mutation in which seizures are one of the main symptoms of the disorder; (iii) infectious origin, as a direct result of a known infection in which seizures are one of the main symptoms. Seizures are a typical symptom in acute infections such as meningitis or encephalitis; (iv) Metabolic origin, in which a metabolic defect causes biochemical changes throughout the body that give rise to epileptic seizures, such as porphyria, uremia, aminoacidopathies, or pyridoxine‐dependent seizures; (v) immune origin, also called autoimmune epilepsies. An immune etiology can be defined in cases where there is evidence of antibody‐mediated central nervous system inflammation; and (vi) unknown origin, in which the cause of epilepsy is not yet known. In this category, it is not possible to establish a specific diagnosis.^8^

## 2 CURRENT TREATMENT FOR EPILEPSY

Due to the complex etiopathogenesis of epilepsy, there are numerous options for its therapeutic approach. Antiseizure medications (ASM) can be classified according to their biopharmaceutical properties or their mechanism of action. At the beginning of the 20th century, the first ASM (phenobarbital, valproate, carbamazepine, benzodiazepines, and phenytoin) began to be used. It was not until the 1990s that second‐generation ASM (gabapentin, pregabalin, lamotrigine, vigabatrin, tiagabine, zonisamide, felbamate, levetiracetam, topiramate, oxcarbazepine, etc) emerged as adjuncts to first‐generation ASM in the clinical practice. Despite the fact that these new ASM achieved an improvement in pharmacokinetics and a decrease in adverse effects compared to those of the first generation, their narrow therapeutic margin and their multiple interactions continue to be a limitation in their use.^5^, ^9^ Due to this fact, the third‐generation ASM (lacosamide, rufinamide, ezogabine, eslicarbazepine, and perampanel) emerged with a more favorable pharmacokinetic profile and increased control of neuronal excitability to prevent epileptic seizure.^10^

In general, their mechanism of action is focused on the decrease or modulation of neuronal excitability,^11^ which in turn stops the expansion of electrical discharges at the brain level.^12^ The mechanism of action of ASM is related to different therapeutic targets and therefore they can be classified in different groups^13^ (Table 1).

**TABLE 1:** Classification of antiseizure medications (ASM) (adapted from Ref. [13])

Mechanism of action	ASM
Glutamate inhibitors	Topiramate (TPM), Phenobarbital (PB), Felbamate, Sodium Valproate (VPA), Levetiracetam (LEV)
Ca channel blocker	TPM, Lamotrigine (LTG), PB, VPA, Gabapentin (GBP), Pregabalin (PGB), LEV, Carbamazepine (CBZ), Oxcarbazepine (OXC), Zonisamide (ZNS), Ethosuximide (ESM)
Na channel blocker	TPM, LTG, VPA, GBP, PGB, CBZ, OXC, Lacosamide, ZNS, Rufinamide
K channel activators	LEV, OXC
GABA agonists	TPM, PB, VPA, GBP, PGB, LEV, Stiripentol, Vigabatrin
SV2A binding	LEV, Brivaracetam
Carbonic anhydrase inhibitors	TPM, ZNS, Sulthiame, acetazolamide
α2δ binding	VPA, ZNS, ESM

Firstly, some ASM act on the excitatory synapse, modulating inotropic glutamate receptors such as AMPA or NMDA receptors, by reducing or blocking their activity. Secondly, other ASM are capable of acting on the ion channels of the extrasynaptic membrane with dependent voltages such as Ca^2+^, Na^+^, or K^+^ channels. In general, these channels play an important role in the spread control of the epileptic crisis as well as in the stabilization and restoration of the physiological conditions of the neuronal membrane. Thirdly, some ASM can act on the inhibitory synapses, reducing the neuronal excitability by acting on the GABAergic system. Finally, the miscellanies group can be found, including ASM with diverse mechanisms of action.^12^, ^13^

As can be observed in Table 1, the majority of ASM possess different mechanisms of action. Due to this fact, monotherapy is preferred in epilepsy treatment and especially among the elder population, thus avoiding possible interactions derived from polymedication.

Moreover, ASM drugs have a narrow therapeutic margin as well as numerous adverse effects. If the pharmacological treatment is not effective, there are other therapeutic alternatives such as electrostimulation of the vagus nerve or deep brain stimulation by means of devices that are implanted in the patient's body. Finally, other option that have been reported to be helpful is the ketogenic diet, which has been described to be able to reduce the number of epileptic seizures, especially in children population.^14^, ^15^, ^16^

## 3 OVERCOMING THE BLOOD‐BRAIN BARRIER IN DRUG‐RESISTANT EPILEPSY

The blood‐brain barrier (BBB) constitutes a physical and metabolic barrier, which controls the BBB exchange of nutrients and xenobiotics and protects the brain microenvironment.^17^, ^18^ Moreover, the permeability of the BBB is one of the most relevant factors that determines drugs bioavailability and ASM resistance. In this area, two main hypotheses describing ASM resistance exist: the target theory and the transporter theory. The first one is based on the fact that molecules targeted by the ASM suffer from modifications that lead to a reduction of ASM therapeutic efficacy. The latter hypothesis claims that there is an aberrant functioning of multidrug transporters and it causes a decrease on the effective brain ASM concentration.^19^, ^20^ For this reason, nanoparticles (NP) constitute an excellent alternative to overcome the BBB achieving therapeutic ASM concentrations. Some types of NP are able to cross the BBB and consequently increase the concentration of drugs in the CNS.^21^, ^22^ Although NP penetration mechanisms are diverse, in general they can be classified into 2 main routes. Firstly, those mechanisms mediated by active transport such as endocytosis (both by adsorption and mediated by receptors such as the lactoferrin receptor) or penetration through of transporters such as glucose transporter 1 (GLUT 1).^23^ The second type of mechanisms involves passive transport mediated by diffusion through endothelial cells.^24^ Furthermore, it has also been reported that NP can also increase ASM concentration either inside the epithelial cells that form the BBB or on the luminal surface, which will favor an increase in drug penetration to the brain.^25^, ^26^

## 4 CONTROLLED DRUG DELIVERY SYSTEMS

Controlled drug delivery systems (CDDS) are pharmaceutical forms aimed to carry different drugs, protect them, target them to a specific organ and release their content in a sustained way to improve the bioavailability and effectiveness of the carried molecule. The first generation (1G) of CDDS was focused on controlling the release kinetics. The second generation (2G) of CDDS produced intelligent polymers, sensitive to the environment, with zero‐order kinetics and depot formulations aimed for long‐lasting effects. Finally, the third generation (3G) was focused on developing carriers with more predictable kinetics through in vitro methods, obtaining highly selective, longer‐lasting and easy to administer CDDS, and, at the same time, overcoming the physicochemical and biological barriers that were not breached by 2G CDDS.^27^

The main properties that a carrier should possess to be a CDDS are as follows: (i) release of the drug at a predetermined rate; (ii) can be administered locally or systemically; (iii) remain in the body for a specified period of time; and (iv) target the carried drug to the specific site of action. Furthermore, the main mechanism that governs the controlled release of a drug is diffusion, due to Fick's law, but drugs can also be released through erosion, swelling, or desorption mechanisms. This release also depends on the aqueous solubility, ionization, PKa, stability, partition coefficient, and molecular weight of the drug.^27^, ^28^, ^29^

Among the most important CDDS developed for nanomedicine, liposomes, micelles, nanoparticles, carbon nanotubes, graphene sheets, hydrogels, dendrimers, polyelectrolyte complex, and quantum dots can be found.

## 5 BIODEGRADABLE NANOPARTICLES FOR EPILEPSY

In recent decades, biodegradable nanoparticles are gaining attention as therapeutic strategies for epilepsy management. NP can cross the BBB, improve selectivity to the brain decreasing side effects, and offer a sustained drug delivery. Biodegradable nanomaterials can be naturally degraded into non‐toxic bioproducts in the body and can be designed for their degradation once arrived to the target site while remaining stable at off‐target places.^30^, ^31^ The two main types of biodegradable NP will be summarized in the following sections along with their applications for epilepsy treatment (Table 2).

**TABLE 2:** Selected relevant preclinical studies with biodegradable nanoparticles for epilepsy disorders

Drug loaded	Type of nanoparticle	Matrix composition	Admin. route	Results	Ref
Alprazolam	Lipid nanoparticle (SLN)	Glyceryl monostearate	iv i.n.	Higher concentration in the brain in intranasal administration.	54
				Bioavailability increased due to the SLN, using a lower dose.	
Carbamazepine	Lipid nanoparticle (SLN)	Phospholipon R80H	v.o.	With MES method, they obtained better anticonvulsant activity after the treatment of SLN with chitosan.	56
				With INH method, they achieved better activity with the SLN without chitosan.	
	Lipid nanoparticle (NLC and SLN)	Lipid myristyl myristate	i.n.	The formulation was incorporated in a thermosensitive mucoadhesive gel.	59
		Cetyl esters wax NF			
				NLC considerably protected the animals against chemically induced convulsions.	
		Crodamol^®^ GTCC‐LQ			
	Polymeric nanoparticle	PLGA	iv	30 times greater efficacy compared to the free drug.	41
				The no influence of the PgP porter on the encapsulated CBZ.	
Catechin hydrate	Polymeric nanoparticle	PLGA‐Chitosan	i.n.	Decrease in the necessary dose of catechin hydrate.	47
				Improvement significantly in brain biodistribution.	
				When administered i.n. the first‐pass hepatic metabolism was avoided.	
Clonazepam	Lipid nanoparticle (SLN and NLC)	Glycerol monostearate	—	The formulation was incorporated in a thermosensitive mucoadhesive gel.	61
		Glyceryl monooleate			
				NLC considerably protected the animals against chemically induced convulsions.	
		Glyceryl behenate			
		Oleic acid			
Curcumin	Lipid nanoparticle (SLN)	Stearic acid	—	Neuroprotective efficiency against oxidative damage.	55
		Lecithin			
				Downregulation of p‐P38 MAPK and apoptosis related proteins.	
Diazepam	Polymeric nanoparticle	PLGA	—	Obtaining NP that can encapsulate diazepam as an ASM.	48
EGCG	Polymeric nanoparticle	PLGA‐PEG	i.n.	Neuroprotective effect.	45
				Anticonvulsant efficacy decreased neuroinflammation and neuronal death.	
Lamotrigine	Lipid nanoparticles (NLC)	Glyceryl monostearate Oleic acid	i.n. v.o.	IN administration maintained the effect of LMT with higher brain concentration. Increased residence time of drug in brain as compared to IN and oral administration.	58
				Higher protective effect of IN administration compared to oral administration with lower doses	
Oxcarbazepine	Polymeric nanoparticles	PLGA	i.n.	Neuroprotective effect.	40
				Reduction of the dosage regimen while maintaining the anticonvulsant activity.	
				Accumulation in cerebral tissue induced model.	
				compatibility with neuronal cells of this novel drug administration system	
Piperine	Polymeric nanoparticles	PLGA‐functionalized with copper oxide quantum dots coated hyaluronic acid	i.n.	Anticonvulsant efficiency in PTZ‐	43
	Polymeric nanoparticles	Chitosan‐STTP	i.p.	Improvement in neuroprotection	44
				Improvement in anticonvulsant activity and activation of astrocytes in epilepsy models compared to free piperine.	
TRH analogues (NP‐355 and NP‐647)	Polymeric nanoparticles	PLGA‐Chitosan	i.n.	Anticonvulsant efficacy decreased neuronal damage.	42
				Biodistribution in brain tissue of NP loaded with THR analogs	
Valproic acid	Lipid nanoparticle (NLC)	Cetyl palmitate	i.p. i.n.	Higher concentration in the brain in intranasal administration.	57
		Soy lecithin			
		Octyldodecanol			
				The same protective effect as systemic administration was observed with lower doses	

### 5.1 Polymeric nanoparticles

Currently, polymeric nanoparticles (PNP) are one of the most widely used CDDS, due to their multiple advantages.^32^ As its name suggest, these nanocarriers are composed by a polymeric structure and range from 10 to 1000 nm. Depending on the polymer composition, which can be natural or synthetic, PNP can have both positive and negative surface charges. This characteristic significantly conditions their biological behavior, muco‐adhesiveness and cell penetration. In addition, PNP can be formulated either as nanocapsules or nanospheres. Nanocapsules possess a vesicular structure, in which drugs are dissolved in a liquid core surrounded by the polymeric capsule. In contrast, nanospheres are composed by a polymeric matrix, in which drugs are dispersed in the matrix gaps or adsorbed onto the spherical surface. The latter PNP have the ability to encapsulate higher amounts of drug.^33^, ^34^

In addition, due to their physicochemical characteristics, PNP can cross physiological barriers such as BBB, allowing to improve the bioavailability and biodistribution of drugs.^35^ At the same time, they allow control of drug release kinetics and, conversely, extend over time their therapeutic action.^36^, ^37^ Additionally, PNP can protect drugs from enzymatic degradation as well as from the action of the immune system.

**FIGURE 1:**
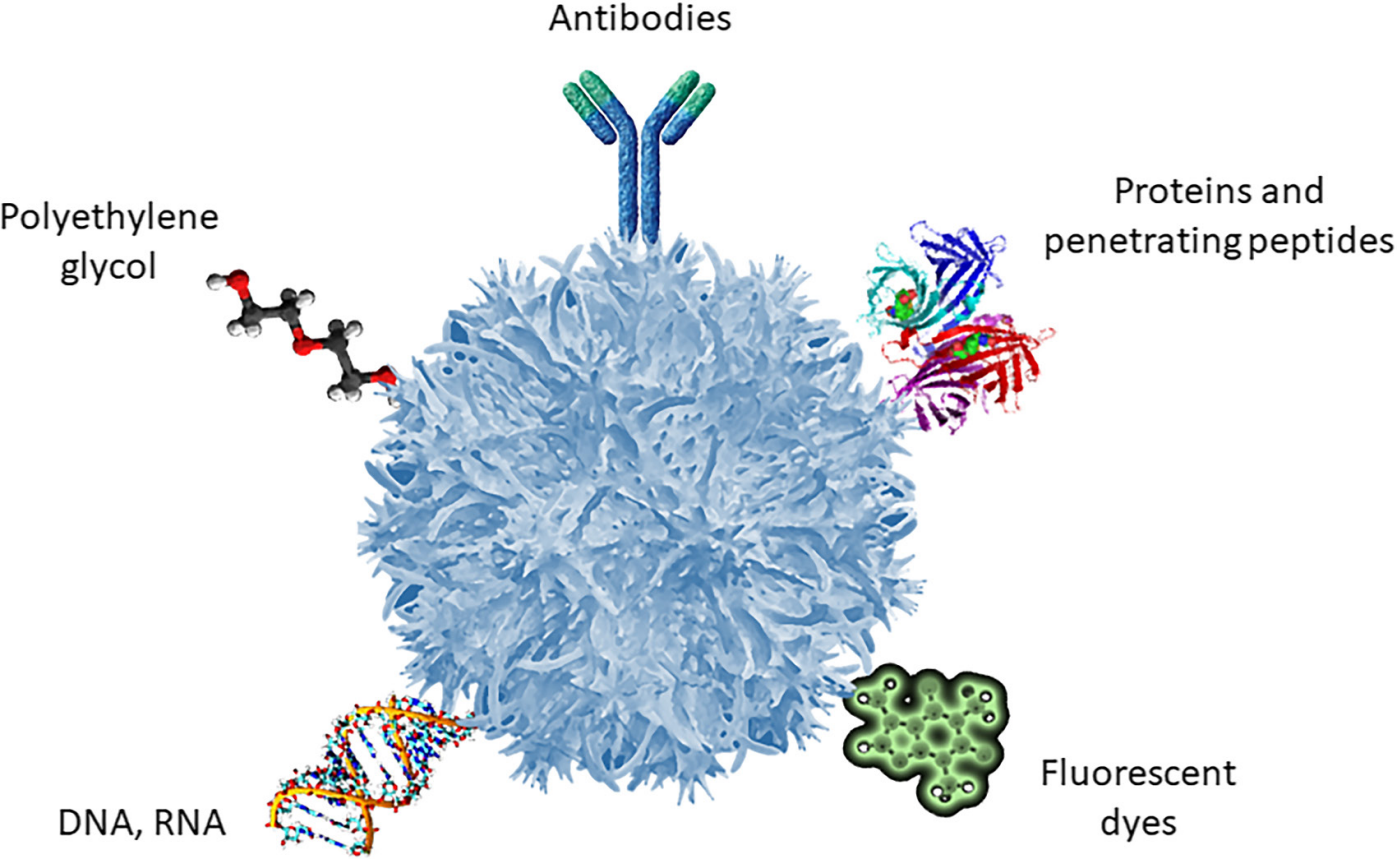
Common ligands of surface functionalization of polymeric nanoparticles

The properties of PNP are given, mainly, by the polymeric matrix material. This matrix can be formed by very diverse polymers or combinations of polymers. The polymers most widely used to manufacture PNP are polylactide (PLA), poly(lactide‐co‐glycolide) (PLGA), chitosan, polyethylenimine (PEI), poly‐ε‐caprolactone (PCL), and Chitosan, all of which gave been approved by the FDA for biomedical applications.^38^, ^39^ In addition, PNP allow the polymeric matrix surface functionalization to guide them selectively to its therapeutic target (Figure 1).

In order to improve the pharmacological treatment of epilepsy and reduce the number of patients with resistance to ASM and their adverse effects, in recent years, many efforts have been invested in order to be able to administer these drugs carried by PNP. An example of this is the study carried out by Musumeci et al where they developed PNP for the intranasal (IN) administration of oxcarbazepine (OXC). These NP were formed by a polymeric matrix of PLGA, in which the OXC was encapsulated. They demonstrated that PLGA NP were capable of reducing the administration posology up to a single dose every 24 hours, reducing multiple administrations and maintaining the epileptic seizures controlled. In addition, they demonstrated the accumulation of these PNP in the brain after IN administration and a neuroprotective effect.^40^

Another interesting study where PLGA NP were developed was carried out by Zybina et al^41^ In this research, the authors managed to encapsulate carbamazepine (CBZ) in PNP. These NP demonstrated that encapsulated CBZ was 30 times more effective against seizures than free CBZ. In addition, they also demonstrated how CBZ encapsulation into PNP was able to overcome the drug resistance mediated by P‐glycoprotein (Pgp). In this sense, Pgp is one of the main transporters that limits drug entry into the brain. Since Pgp is responsible for the resistance of some patients to treatment with ASM, it is highly interesting that PLGA NP demonstrated to overcome this obstacle.^41^

Moreover, the use of some therapeutic compounds against epilepsy is limited not only by the lack of efficacy but also by their low bioavailability and high degradability by body enzymes. These makes some drugs unable for the treatment of CNS pathologies. An example of this is thyrotropin‐releasing hormone (TRH) analogues called NP‐355 and NP‐647. In this area, Kaur et al^42^ developed chitosan‐coated PLGA PNP capable of encapsulating these TRH analogues for IN administration with high mucoadhesive properties. They not only demonstrated that TRH PNP were capable of reaching the brain after IN administration, but also in in vivo studies demonstrated that TRH NP significantly inhibited seizures and neuronal death in the pentylenetetrazole (PTZ) ‐induced seizures model.^42^ Using the same in vivo model, piperine‐loaded PNP functionalized with copper oxide quantum dots and coated with hyaluronic acid proved also a significant anticonvulsant effect after IN administration.^43^ In addition, piperine has also been encapsulated in chitosan PNP for IP administration showing increased neuroprotectant properties against free piperine.^44^

Furthermore, Cano et al^45^ developed PNP capable of encapsulating epigallocatechin‐3‐gallate (EGCG), one of the most abundant catechins in tea. EGCG is known to reduce seizure episodes, but the exact mechanism is not yet fully elucidated.^38^, ^46^ EGCG PNP were developed using a PLGA matrix along with polyethylene glycol (PEG) functionalization. EGCG PNP were assessed in an in vivo study using the kainic acid‐induced mouse temporal lobe epilepsy model showing interesting results. EGCG PNP provided increased efficacy against convulsive episodes and also contributed to a greater decrease in neuroinflammation and neuronal death compared to the administration of non‐encapsulated EGCG.^45^ Moreover, in another study, catechin hydrate was also encapsulated into PLGA NP. In this case, they were coated with chitosan providing positive surface charge. After IN administration, PLGA‐Chitosan PNP demonstrated an improved bran distribution and were able to avoid hepatic metabolism.^47^

Other studies such as the one carried out by Bohrey et al^48^ developed PNP with therapeutic potential for the treatment of epilepsy. The authors were able to encapsulate diazepam, which is used to treat seizures during the epileptic episode. However, in this study, in vitro and in vivo would be necessary to confirm that diazepam loaded PNP are effective and can cross the BBB since their size is greater than 200 nm and this may difficult their BBB penetration.^48^ Moreover, the study carried out by Lopalco et al^49^ developed a formulation of PNP encapsulating OXC and using PLGA as a polymer as a therapeutic tool to treat epilepsy in pregnant women. In this particular scenario, increased efforts should be undertaken toward an optimization of the formulation in order to obtain a targeted drug delivery carrier specially focused on pregnant population.^49^

### 5.2 Lipid nanoparticles

Among nanoparticulate drug delivery systems, lipid NP (LNP) constitute one of the most versatile due to their low toxicity and great drug loading capacity. Due to the structural similarity between the lipids used to fabricate NP (in many cases from natural origin) and the lipids of the endothelial cells of BBB, the passage of these nanocarriers by the transcellular pathway is favored.^34^ Furthermore, their high biocompatibility and low immunogenicity allow them to be rendered as the best candidates for targeting diseases of the CNS.^50^

There are two main types of LNP, solid lipid nanoparticles (SLN) and the second generation of lipid nanoparticles, nanostructured lipid carriers (NLC). NLC present enhanced stability, higher drug loading capacity and are able to prevent drug expulsion during storage.^51^ Several studies have reported anticonvulsant activity on different formulations of SLN and NLC, which are summarized below. Moreover, SLN and NLC are the two most widely studied lipid‐based nanocarriers for brain pathologies, since they have the physicochemical potential to deliver drugs in the deepest brain regions.^50^

#### 5.2.1 Solid lipid nanoparticles

**FIGURE 2:**
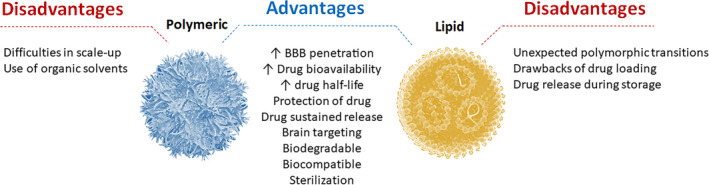
Main advantages and disadvantages of both polymeric and lipid nanoparticles

By definition, SLN are colloidal drug delivery systems with a solid structure at body temperature with an average size comprised between 40 nm and 1 µm, composed by a biodegradable and biocompatible lipid matrix stabilized by a suitable surfactant.^52^, ^53^ Therefore, the advantages of SLN as pharmaceutical drug delivery carriers comprise the use of safe recognized excipients (which not occur with the organic solvents used in the fabrication of PNP), thus presenting an excellent toxicity and tolerability; protection of the incorporated chemical compounds; high physical stability compared to liposomes; capability of incorporation both lipophilic and hydrophilic molecules; sustained release of the drugs; targeting mechanisms; easy fabrication process; and reproducible scale up for industrial formulation development. However, SLN also present some important disadvantages, such unexpected dynamics of polymorphic transitions, drawbacks of low drug loading capacity, caused by the crystalline structure of the solid lipid, and undesirable drug release during the storage (Figure 2).^50^

Several studies have been carried out using SLN for epilepsy treatment. In this sense, an excellent study was carried out by Singh et al^54^ where the authors investigated the biodistribution of alprazolam (APZ)‐loaded SLN. SLN were formed by a biodegradable lipid, glyceryl monostearate. Their biodistribution was studied in rats coupling APZ and APZ‐loaded SLN with a radioactive tracer. The formulations were administered by IN and intravenous (IV) routes. The authors observed that the concentration in the brain was found to be significantly higher after IN than IV administration. Secondly, they observed that the concentration of APZ solution by the IN route remained significantly lower when compared to SLN using the same administration route. Thus, we confirmed that loading APZ in SLN could play a significant role in lowering APZ dose since bioavailability increased. Moreover, SLN formulation showed a higher targeting efficiency compared to the APZ solution. The authors hypothesize that this fact may be due to low‐density lipid receptors on the BBB.^54^

Huang et al^55^ fabricated SLN containing a novel epilepsy potential treatment, curcumin. Curcumin is a natural, antioxidant, and neuroprotectant compound. They developed interesting in vitro experiments, showing the potential of curcumin SLN as ASM. Although the results obtained were very promising, in vivo experiments are required in order to confirm the efficacy of this approach.^55^ In addition, Nair et al^56^ evaluated SLN containing CBZ formed by phospholipon R80H and coated with chitosan. They studied CBZ SLN anticonvulsant activity in rats by administering CBZ solution, CBZ SLN without chitosan, and CBZ SLN with chitosan (dose of 1 mL/100 mg body weight). The therapeutic activity of CBZ SLN with and without chitosan varied depending on the assessments carried out. In the maximal electroshock (MES) method, where an electrical stimulus was applied through ear electrodes, the SLN with chitosan shown better activity. On the other hand, in the isoniazid induced convulsions (INH) method, SLN without chitosan showed better anticonvulsant activity. Although additional assessments will shed more light about this investigation, the results seem to indicate that CBZ SLN with chitosan had a better potential due to their higher encapsulation of CBZ and increased physical stability.^56^

#### 5.2.2 Nanostructured lipid carriers

Nanostructured lipid carriers were developed as a second generation of LNP, aiming to overcome the main limitations of SLN. This type of nanostructures presents a lipid matrix with an internal structure consisting of both liquid and solid lipids at room temperature. The main advantages of NLC over SLN are due to their less organized lipid core and comprised an increased loading capacity (caused by the liquid oil droplets in the solid matrix), strong immobilization of entrapped drugs, and reduced expulsion of loaded molecules during storage due to their low crystallinity index and slower polymorphic transition. However, it is important to remark that both SLN and NLC have lower capacity to entrap hydrophilic drugs than PNP because of their partitioning effects during fabrication. It is well known that the chemical nature of the loaded compounds significantly influences their encapsulation and release from colloidal systems, and it is obvious that lipophilic drugs present a better entrapment efficiency and delivery profile in LNP.^50^

In this area, Eskandari et al evaluated the anticonvulsant activity of sodium valproate (VPA) loaded into NLC after IN or intraperitoneal (IP) administration. VPA NLC were composed by biodegradable lipids such as cetyl palmitate and soy lecithin. They compared the positive control (VPA solution at 150 mg/kg), empty NLC (without VPA), and VPA NLC, after IN and IP administration. The authors determined VPA concentration in plasma and brain tissue. After 60 minutes post‐administration, the higher brain/plasma ratio of VPA was obtained with IN administration of VPA NLC. Moreover, it should also be highlighted that the IN administrated dose was lower than the administrated trough IP route. On the other hand, they studied the neuroprotective effect of VPA. The neuroprotective activity of VPA NLC administrated by IN route were similar to VPA control. This study clearly demonstrated that IN administration of VPA NLC could constitute a potentially suitable method to treat epilepsy.^57^ Moreover, IN administration has also been successfully employed by other authors.^58^

Moreover, Montoto et al^59^ synthetized CBZ loaded in NLC with biodegradable lipids such as lipid myristyl myristate, cetyl esters wax, and Crodamol^®^. In their preliminary study, they injected IP in mice models a CBZ control (CBZ solution 30 mg/kg), two negative controls (physiological solution and empty NLC) and CBZ NLC (30 mg/kg). The results showed that both CBZ control and the CBZ NLC presented anticonvulsant activity until 4 hours. However, after 4 hours, only the CBZ NLC presented therapeutic activity, probably due to prolonged release of CBZ from the NLC core.^59^ Lately, Montoto et al synthetized OXCBZ NLC for an in vitro study. They observed an enhanced permeability of the encapsulated drug compared to the free OXCBZ into MDCK‐MDR1 cells. Also, they concluded that there was not interaction between de NLC with studied immunoglobulins or bovine serum albumin, but this investigation should be confirmed by in vivo studies.^60^

Overall, LNP have a great potential to treat epilepsy since they improve the bioavailability and safety profile of ASM.^56^ An interesting study performed by Montoto et al^59^ compared CBZ loaded into SLN and NLC intraperitoneally administered. However, due to the advantages of NLC over SLN, the authors only studied the in vivo activity of CBZ NLC. Furthermore, their formulation protected of seizures in 1 of every 5 mice. Although the study is highly interesting, it might be possible that the CBZ NLC could exert increased effects using other administration routes, such as IN.^59^ Moreover, NLC dispersion in thermosensitive mucoadhesive gels has also proven to be an advantage in order to obtain an slower drug release profile.^61^

## 6 CONCLUSION

Epilepsy is a neurological disease with high incidence that involves seizure episodes with serious consequences for the well‐being and health of patients. Some of the main problems that treatment with ASM present is the narrow therapeutic margin of these drugs as well as the resistance observed during long‐term treatments. These has led to a high percentage of patients who cannot control seizures with ASM. In this sense, biodegradable NP are postulated as a novel therapeutic alternative for the treatment with ASM in a safer and more efficient way, allowing to reduce adverse effects, improve bioavailability, and increase dose interval. Moreover, NP could be used to increase the bioavailability and specificity of certain medications, thus opening a window for personalized therapeutic treatments.

Although NP could constitute a feasible alternative for the future treatment of epilepsy, there is still a long path to be explored in their development and research for epilepsy. Therefore, one limitation of the present topic is the small number of studies focused on evaluating nanomaterials in experimental models of drug‐resistant seizures or epilepsy. Therefore, it is necessary to increase scientific efforts toward the investigations of effective and safe biodegradable nanocarriers for the treatment of epilepsy.

## 7 FUTURE PERSPECTIVES

Research in the next few years should focus on elucidating the molecular pathways involved in the pathogenesis of refractory epilepsies. Likewise, a better understanding of the BBB complexity, dynamic interface, and physiological changes would help to develop novel strategies to overcome these permeability restrictions and to obtain an efficient CNS drug delivery. In this context, polymeric and lipid nanocarriers are one of the best therapeutic tools to solve these issues. However, scale‐up of the manufacturing process of many nanodevices to industry is nowadays a technological challenge. Likewise, translation of nanocarriers‐based treatments to the clinical practice is currently a complicated and expensive process that pharmaceutical industry and clinics must confront. However, nanotechnology has the potential to overcome all the handicaps of ASM. In this sense, biodegradable NP can be placed in other devices or carriers avoiding direct intravenous administration. In this area, intranasal administration has proven to be especially effective. Moreover, incorporation of NP in transdermal drug delivery systems would also provide a long‐term treatment, with constant effective concentrations, being able to cross the BBB and a greater adherence and acceptability by the patient to the treatment. Furthermore, NP materials vary ASM biological behavior, safety, and biocompatibility, and therefore, validation of in vitro and in vivo protocols is also needed to appropriately evaluate them. Likewise, government's regulatory policies are still unsolved aspects that hinder their access to the market. Thus, the extrapolation of these vehicles to clinical practice remains one of the most ambitious challenges in this field and will be one of the objectives to be achieved in the next decade of most nanomedicine trials.

## CONFLICT OF INTEREST

None of the authors has any conflicts of interest including any financial, personal, or other relationships with other people or organizations. We confirm that we have read the Journal's position on issues involved in ethical publication and affirm that this report is consistent with those guidelines.

## AUTHOR CONTRIBUTIONS

LB and GE performed the conceptualization, bibliographic search, and writing of the original draft. ME, ME, ES, MLG, and AC contributed in the writing/review and editing. AC and ESL contributed in the conceptualization, supervision, writing/review, and editing. All authors have made a substantial contribution to the work. All authors have read and agreed to the published version of the manuscript.
